# Glucose-dependent insulinotropic polypeptide receptor agonism improves heart failure with antifibrosis through Akt-dependent nitric oxide signaling

**DOI:** 10.1186/s12929-026-01288-1

**Published:** 2026-08-31

**Authors:** Ting-Wei Lee, Ting-I Lee, Satoshi Higa, Yu-Hsun Kao, Yi-Jen Chen

**Affiliations:** 1 Division of Endocrinology and Metabolism, Department of Internal Medicine, School of Medicine, College of Medicine, Taipei Medical University, Taipei, 11031 Taiwan; 2 Division of Endocrinology and Metabolism, Department of Internal Medicine, Wan Fang Hospital, Taipei Medical University, Taipei, 11696 Taiwan; 3 Cardiac Electrophysiology and Pacing Laboratory, Division of Cardiovascular Medicine, Makiminato Central Hospital, Makiminato, Urasoe, Okinawa 901-2131 Japan; 4 Graduate Institute of Clinical Medicine, College of Medicine, Taipei Medical University, No.250, Wuxing Street, Taipei, 11031 Taiwan; 5 Department of Medical Education and Research, Wan Fang Hospital, Taipei Medical University, Taipei, 11696 Taiwan; 6 Cardiovascular Research Center, Wan Fang Hospital, Taipei Medical University, Taipei, 11696 Taiwan

**Keywords:** Akt, Fibroblast, Fibrosis, Glucose-dependent insulinotropic polypeptide, Heart failure, Nitric oxide

## Abstract

**Background:**

Cardiac fibrosis is a pathological remodeling process that contributes to the development and progression of heart failure. Although glucose-dependent insulinotropic polypeptide (GIP) may exert antifibrotic effects, how GIP receptor activation regulates cardiac fibrogenesis and modulates cardiac function in heart failure remains unclear. This study investigated whether GIP receptor agonism suppresses cardiac fibroblast activity and improves heart failure and explored the underlying mechanisms by using cellular and animal models.

**Methods:**

Human cardiac fibroblasts were treated with [D-Ala^2^]GIP (DA-GIP; 10, 100, or 300 nM) for 24 h or left untreated (control). Fibroblast migration, collagen production, and intracellular signaling were examined using wound healing, immunoblotting, enzyme-linked immunosorbent, and fluorometric assays. Cardiac structure, function, and fibrosis were assessed through echocardiography and Masson’s trichrome staining in rats with isoproterenol-induced heart failure with and without DA-GIP (24 nM/kg, twice daily for 2 weeks) administration.

**Results:**

Compared with control cells, DA-GIP (300 nM)-treated cardiac fibroblasts exhibited a significantly lower migratory activity and reduced expression levels of pro-collagen IA1, pro-collagen III, and transforming growth factor-β1 proteins. Additionally, DA-GIP increased nitric oxide (NO) production and promoted endothelial NO synthase (eNOS) and protein kinase B (Akt) activation in cardiac fibroblasts. Notably, Akt inhibition blocked DA-GIP-induced eNOS activation, and treatment with *N*^*ω*^-nitro-L-arginine methyl ester (a NO synthase inhibitor, 100 μM) attenuated the antifibrotic effect of DA-GIP. In heart failure rats, DA-GIP reduced myocardial fibrosis, chamber dilatation, and systolic dysfunction.

**Conclusions:**

DA-GIP suppresses cardiac fibroblast activity through Akt-dependent eNOS activation and subsequent NO production, thereby improving cardiac remodeling and function in experimental heart failure.

## Background

Glucose-dependent insulinotropic polypeptide (GIP) and glucagon-like peptide-1 (GLP-1) are two major incretin hormones produced by the gut. They stimulate pancreatic β-cells to increase insulin secretion after food intake. GLP-1 receptor agonists are widely used to treat type 2 diabetes mellitus (DM) because they reduce blood glucose, promote weight loss, and confer cardiovascular benefits [1]. However, the therapeutic potential of GIP receptor agonists was initially overlooked because their insulinotropic effects are markedly attenuated in individuals with hyperglycemia [2, 3]. Nonetheless, recent clinical data indicate that tirzepatide, a dual GIP and GLP-1 receptor agonist, is considerably more effective than GLP-1 receptor agonists alone in reducing blood glucose and body weight [4, 5], highlighting the metabolic benefits of GIP receptor agonists. Although the GIP receptor is mainly expressed in pancreatic islet β-cells, it is abundantly expressed also in human myocardial tissue [1, 6]. A human genetic study demonstrated that sustained GIP signaling exerts beneficial cardiometabolic effects [7]. A low serum level of GIP at hospital admission was associated with an increased risk of cardiovascular mortality in patients with acute myocardial infarction [8]. These findings suggest that modulation of GIP signaling may directly influence cardiovascular health.

Cardiac fibrosis is a pathological remodeling process that commonly occurs during the later stages of heart disease. This process increases myocardial stiffness, disrupts cardiac architecture, and impairs both cardiac relaxation and contraction, ultimately leading to the development of heart failure [9–11]. However, effective therapies for cardiac fibrosis remain limited. Müller et al. demonstrated that mice lacking GIP receptor exhibited increased cardiac fibrosis and age-related systolic dysfunction [2]. Treatment with GIP reduced myocardial fibrosis in angiotensin II-infused, apolipoprotein E-deficient mice [12] and diabetic db/db mice [13], indicating the antifibrotic potential of GIP receptor agonism. In addition, a retrospective cohort analysis study demonstrated that treatment with tirzepatide was associated with a significantly lower risks of heart failure exacerbation and new-onset systolic heart failure than treatment with GLP-1 receptor agonists in patients with type 2 DM and preexisting ischemic heart disease [14]. However, experimental evidence for the beneficial effects of GIP on cardiac function in heart failure remains lacking. Furthermore, whether GIP directly affects cultured cardiac fibroblasts, and if so, how the activation of GIP signaling regulates cardiac fibrogenesis remain unclear.

Data from mice suggest that GIP reduces cardiac fibrosis by downregulating transforming growth factor (TGF)-β [12, 13]. Activation of GIP receptor induced the cyclic guanosine monophosphate (cGMP)/protein kinase G (PKG) pathway in murine cardiomyocytes [15, 16]. cGMP is a widely distributed intracellular second messenger that regulates multiple cardiovascular functions. It is synthesized by soluble guanylyl cyclase in response to nitric oxide (NO) stimulation and by membrane-bound guanylyl cyclase in response to natriuretic peptide activation [17, 18]. Upregulated cGMP/PKG signaling directly inhibited cardiac fibroblast activity in mice by antagonizing the TGF-β1 pathway [19, 20]. Furthermore, NO, which is synthesized from L-arginine by nitric oxide synthase (NOS), also exerts antifibrotic effects during cardiac remodeling. Pharmacological inhibition of NOS exacerbated angiotensin II-induced myocardial fibrosis in rats [21]. Although NO suppresses cardiac fibrosis mainly by activating the cGMP/PKG pathway [22], treatment with NO donors inhibited cell proliferation in mouse embryonic fibroblasts lacking soluble guanylyl cyclase activity [23]. Therefore, NO may exert its antifibrotic effects through cGMP-independent mechanisms. However, whether GIP receptor activation directly regulates cardiac fibroblast activity and whether its potential antifibrotic effects are mediated through the NO/cGMP/PKG pathway remains to be elucidated. In the present study, we investigated the effects of GIP receptor agonism on cardiac fibrogenesis in cultured cardiac fibroblasts and in a rat model of heart failure and explored the underlying mechanisms.

## Materials and methods

### Cell culture

Human cardiac fibroblasts derived from human atrial tissue were purchased from Lonza Research Laboratory (Walkersville, MD, USA). The cells were seeded as monolayers on uncoated culture dishes and maintained in FGM-3 Cardiac Fibroblast Growth Medium-3 BulletKit (Lonza; supplemented with 14.999 mM HEPES, 14.010 mM sodium bicarbonate, and 10% fetal bovine serum) at 37 °C in a humidified incubator with 5% CO_2_. Fibroblasts from passages 4–6 were used to minimize functional variability. Because native GIP has a short circulating half-life (5–7 min), [D-Ala^2^] GIP (DA-GIP)—a degradation-resistant analog with receptor binding and activation properties similar to those of native GIP [24]—was used for both in vitro and in vivo experiments. Human cardiac fibroblasts were cultured without any treatment (control) or treated with DA-GIP (10, 100, or 300 nM; Tocris Bioscience, Bristol, UK) for 24 h in the presence or absence of KT5823 (PKG inhibitor; 1 μM; Tocris Bioscience), SQ22536 (adenylyl cyclase inhibitor; 100 μM; Tocris Bioscience), A6730 (Akt inhibitor; 3 μM; Sigma-Aldrich), or *N*^*ω*^-nitro-L-arginine methyl ester (L-NAME; NOS inhibitor; 100 μM; Sigma-Aldrich, St. Louis, MO, USA). Then, the cells were harvested for subsequent experiments.

### Fibroblast migration assay

A wound healing assay was performed to assess the migration of cardiac fibroblasts. In brief, human cardiac fibroblasts were cultured in six-well plates and treated with DA-GIP (10, 100, or 300 nM) in the presence or absence of KT5823 (1 μM), SQ22536 (100 μM), or L-NAME (100 μM) for 24 h. A linear scratch was created using a P200 pipette tip at 8 h before the end of the treatment period. The wound area was measured at 0 and 8 h by using ImageJ 1.54g (National Institutes of Health, Bethesda, MD, USA). Net cell migration was calculated as the reduction in the wound area relative to baseline and is expressed as the fold change relative to the results of the control group.

### Immunoblotting

Control fibroblasts and fibroblasts treated with DA-GIP, A6730 or L-NAME were lysed using M-PER Mammalian Protein Extraction Reagent (Thermo Fisher Scientific, Waltham, MA, USA) supplemented with Halt Protease and Phosphatase Inhibitor Cocktail (Thermo Fisher Scientific), as described previously [25]. Protein concentrations were measured using the Qubit Protein Assay (Thermo Fisher Scientific). Equal amounts of protein were separated through sodium dodecyl sulfate polyacrylamide gel electrophoresis under reducing conditions. The resultant protein bands were transferred onto polyvinylidene difluoride membranes (Amersham Biosciences, Buckinghamshire, UK). The membranes were incubated with primary antibodies against pro-collagen IA1 (dilution: 1:500; Santa Cruz Biotechnology, Santa Cruz, CA, USA), pro-collagen III (dilution: 1:2,000; Abcam, Cambridge, UK), α-smooth muscle actin (α-SMA; dilution: 1:8,000; Abcam), TGF-β1 (dilution: 1:1,000; Cell Signaling Technology, Danvers, MA, USA), endothelial NOS (eNOS; dilution: 1:1,000; Cell Signaling Technology), phosphorylated eNOS (dilution: 1:1,000; Cell Signaling Technology), neuronal NOS (nNOS; dilution: 1:3,000; Thermo Fisher Scientific), phosphorylated nNOS (dilution: 1:3,000; Thermo Fisher Scientific), inducible NOS (iNOS; dilution: 1:6,000; Thermo Fisher Scientific), Akt (dilution: 1:2,000; Cell Signaling Technology), and phosphorylated Akt (dilution: 1:3,000; Cell Signaling Technology). Horseradish peroxidase–conjugated secondary antibodies (Leinco Technology, St. Louis, MO, USA) were then applied, and protein bands were detected using an enhanced chemiluminescence system (Millipore, Darmstadt, Germany). Densitometric analyses were performed using AlphaEaseFC (Alpha Innotech, San Leandro, CA, USA). Protein levels were normalized to those of β-actin (dilution: 1:8,000; Antibodies.com, Cambridge, UK) to confirm equal protein loading.

### Cell proliferation assay

A 5-ethynyl-2*′*-deoxyuridine (EdU) incorporation assay was performed using the Click-iT Plus EdU Flow Cytometry Assay Kit (Thermo Fisher Scientific), as described previously [26]. Control and DA-GIP (10, 100, or 300 nM for 24 h)-treated fibroblasts were incubated with Click-iT EdU (10 μM), washed with 1% bovine serum albumin in phosphate-buffered saline, and then fixed and permeabilized. The cells were subsequently incubated with 0.5 mL of Click-iT Plus reaction cocktail for 30 min to detect EdU incorporation. After staining, the cells were resuspended in 1 × Click-iT saponin-based permeabilization and wash reagent and analyzed through flow cytometry.

### TGF-β1 enzyme-linked immunosorbent assay

TGF-β1 level in cardiac fibroblast culture supernatants was measured using an enzyme-linked immunosorbent assay kit (R&D Systems, BioTechne, Minneapolis, MN, USA). In accordance with the manufacturer’s instructions, the supernatants were acidified with 20 μL of 1 N HCl and incubated at room temperature for 10 min to activate latent TGF-β1, followed by neutralization with 20 μL of 1.2 N NaOH/0.5 M HEPES. Absorbance was measured at 450 nm, and the TGF-β1 level was normalized to the total protein content of the corresponding fibroblast sample.

### Measurement of intracellular cGMP levels

Control and DA-GIP (300 nM)-treated fibroblasts were homogenized in 0.1 M HCl at room temperature for 20 min. The homogenates were centrifuged at 1000 × *g* for 10 min, and the supernatants were collected. Intracellular cGMP levels were quantified using a commercially available enzyme-linked immunosorbent assay kit (Cayman Chemical Co., Ann Arbor, MI, USA), following the manufacturer’s instructions. cGMP level was normalized to the total protein content of corresponding fibroblast sample.

### Measurement of NO levels

Accumulation of total NO metabolites (nitrite and nitrate) in the culture supernatants of control and DA-GIP (300 nM)-treated fibroblasts was measured using a commercial fluorometric NO assay kit (Abcam). In brief, nitrate was enzymatically converted to nitrite by nitrate reductase, followed by reaction with the fluorescent probe 2,3-diaminonaphthalene. The resulting fluorescence intensity was proportional to the total NO production. Each sample was collected from 1 × 10^6^ cells, and NO level was normalized to cell count.

### Effects of DA-GIP on heart failure rats

Male Wistar rats (age: 8 weeks; BioLASCO Taiwan, Taipei, Taiwan) were housed in the animal facility of Taipei Medical University under standard laboratory conditions (temperature: 20–22 °C; photoperiod: 12-h light–dark cycle) with ad libitum access to food and water. After a 2-week acclimatization period, heart failure was induced in 10 rats (age: 10 weeks) through a subcutaneous injection of isoproterenol (100 mg/kg; Sigma) once a week for 2 weeks. The rats with heart failure were then randomly assigned to receive either DA-GIP (24 nM/kg body weight, administered subcutaneously twice a day; *n* = 5) or vehicle (0.9% normal saline; *n* = 5) for 2 weeks. All animal experiment protocols adhered to the guidelines issued by the Institutional Animal Care and Use Committee of Taipei Medical University and were approved by the local animal ethics review board (LAC2023-0458).

Echocardiography was performed to examine cardiac structure and function in age-matched control rats, vehicle-treated heart failure rats, and DA-GIP-treated heart failure rats. For this, the rats were anesthetized with 2% isoflurane in oxygen, placed in the left lateral decubitus position, and examined using a Vivid i cardiovascular ultrasound system (GE Healthcare, Haifa, Israel) equipped with a 10-MHz transducer (imaging depth: 2.5 cm; frame rate: 225 frames/s) [27]. Rats with a left ventricular (LV) fractional shortening of <45% were classified as having heart failure [27]. Blood samples were collected after the 2-week treatment period. Plasma insulin levels were measured using the Rat Insulin Enzyme Immunoassay Kit (Bertin Pharma, Montigny-le-Bretonneux, France). Serum biochemical parameters were analyzed using SPOTCHEM II reagent strips. The rats were subsequently euthanized by overdose with 5% isoflurane in oxygen, and their hearts were harvested for histological examination.

### Assessment of LV fibrosis

Myocardial fibrosis was evaluated as described previously [27]. In brief, LV tissues were fixed with 4% formaldehyde, embedded in paraffin, and stained with Masson’s trichrome. Fibrotic areas were stained blue, whereas cardiomyocytes were stained red. Bright-field images were acquired and analyzed using ImageJ 1.54 g. Fibrosis was quantified as the collagen volume fraction, calculated as the ratio of the total collagen-stained area to the total LV tissue area.

### Statistical analysis

All quantitative data are expressed as mean ± standard error of the mean values. Statistical comparisons among fibroblasts under different experimental conditions were performed using one-way analysis of variance (ANOVA), repeated-measures ANOVA, or ANOVA on ranks, followed by Tukey’s post hoc test where appropriate. Paired *t* tests were used for paired comparisons. A *p* value of <0.05 indicated statistical significance.

## Results

### GIP receptor agonism inhibits cardiac fibroblast activity

**Fig. 1:**
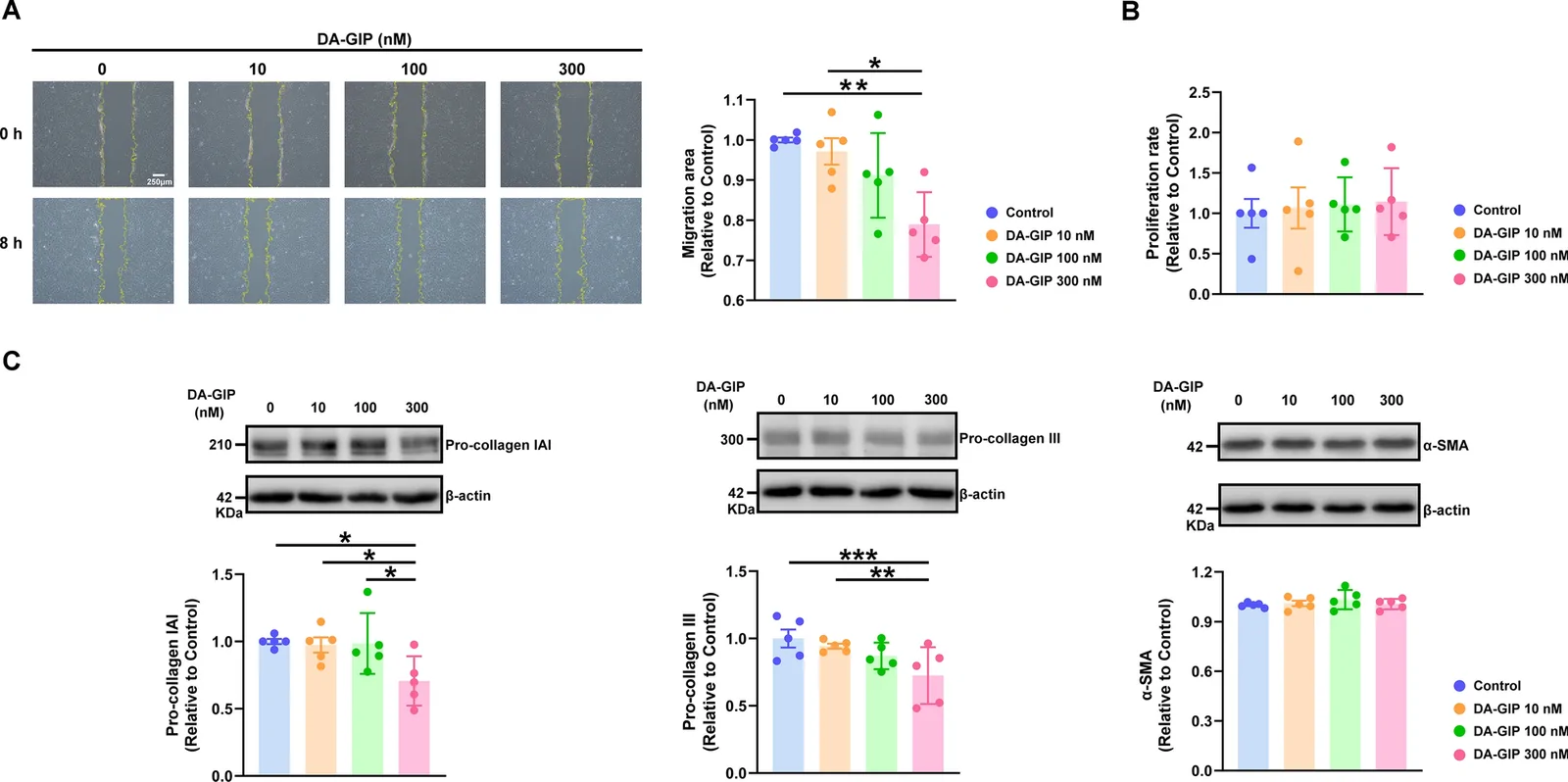
Effects of DA-GIP on human cardiac fibroblast migration, proliferation, collagen production, and α-smooth muscle actin (α-SMA) expression. **A** Representative images (left panel) and quantitative data (right panel, *n* = 5) from a wound healing assay assessing cardiac fibroblast migration. Confluent fibroblasts were scratched at 8 h before the end of DA-GIP treatment. Images were captured at 0 h and 8 h after wounding in control and DA-GIP (10, 100, or 300 nM)-treated cardiac fibroblasts. Scale bar: 250 μm. **B** Quantitative data (*n* = 5) for fibroblast proliferation (assessed using a 5-ethynyl-2′-deoxyuridine incorporation assay) in control and DA-GIP (10, 100, or 300 nM)-treated cardiac fibroblasts. **C** Representative immunoblots and quantitative data for pro-collagen IA1, pro-collagen III, and α-SMA expression in control and DA-GIP (10, 100, or 300 nM)-treated cardiac fibroblasts (*n* = 5). ^*^p < 0.05, ^**^p < 0.01, and ^***^p < 0.005

DA-GIP (300 nM)-treated human cardiac fibroblasts exhibited significantly lower migratory activity than did control fibroblasts. By contrast, control fibroblasts and fibroblasts treated with DA-GIP at 10 or 100 nM had similar migratory activity (Fig. 1A). The EdU incorporation assay revealed similar proliferation rates among control and DA-GIP (10, 100, or 300 nM)-treated fibroblasts (Fig. 1B). Fibroblasts treated with 300 nM DA-GIP, but not 10 or 100 nM DA-GIP, exhibited significantly lower expression levels of pro-collagen IA1 and pro-collagen III proteins than did control fibroblasts (Fig. 1C). However, α-SMA protein expression levels were similar among control, DA-GIP (10 nM)–treated, DA-GIP (100 nM)–treated, and DA-GIP (300 nM)–treated fibroblasts (Fig. 1C).

### GIP receptor activation regulates TGF-β1 and cGMP levels in human cardiac fibroblasts

**Fig. 2:**
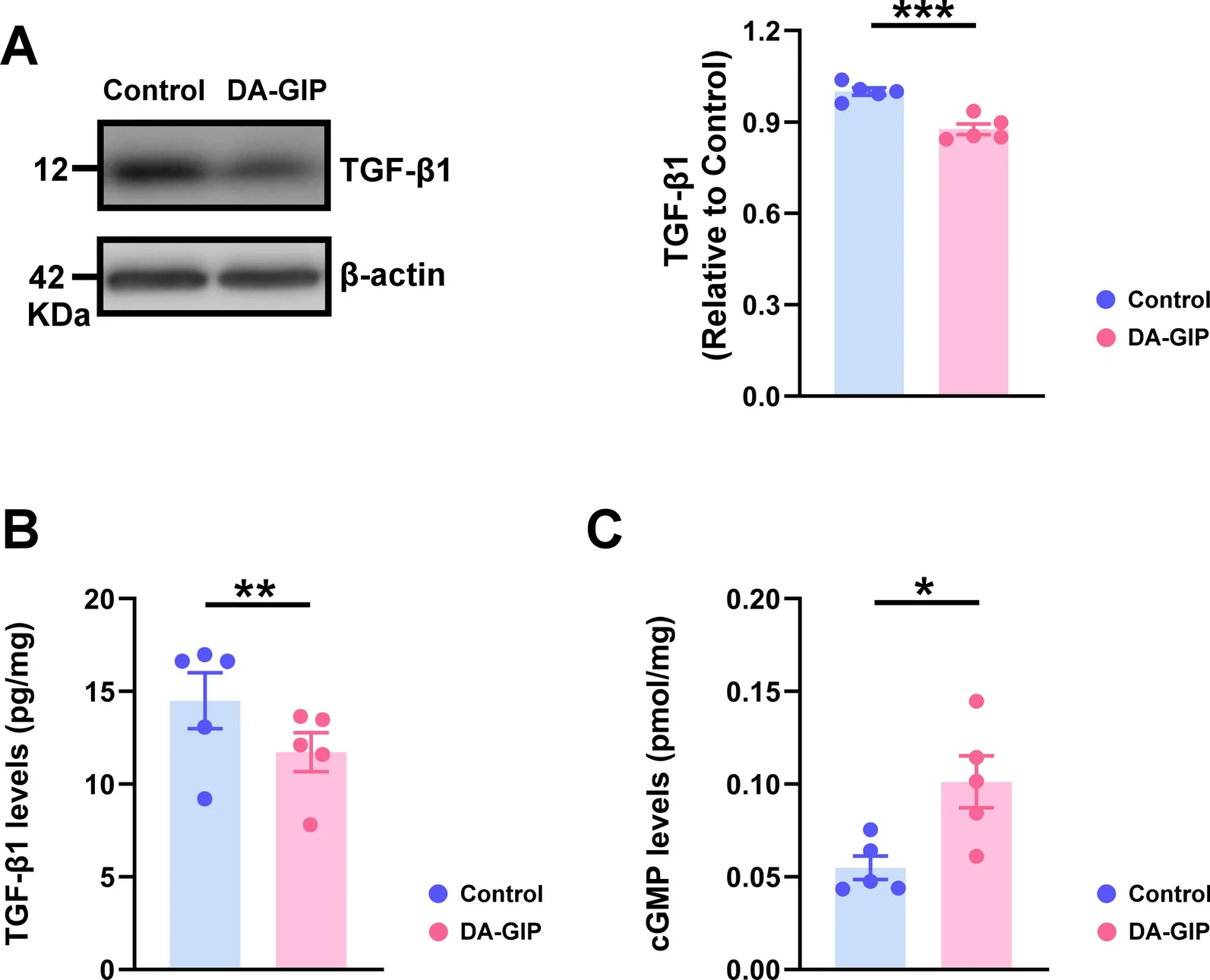
DA-GIP downregulates transforming growth factor (TGF)-β1 expression and increases cyclic guanosine monophosphate (cGMP) levels in human cardiac fibroblasts. **A** Representative immunoblots and quantitative data (*n* = 5) for TGF-β1 expression in control and DA-GIP (300 nM)-treated cardiac fibroblasts. **B** TGF-β1 levels in the culture supernatants of control and DA-GIP (300 nM)-treated cardiac fibroblasts (*n* = 5). **C** Quantitative data (n = 5) for intracellular cGMP levels in control and DA-GIP (300 nM)-treated cardiac fibroblasts. ^*^p < 0.05, ^**^p < 0.01, and ^***^p < 0.005

**Fig. 3:**
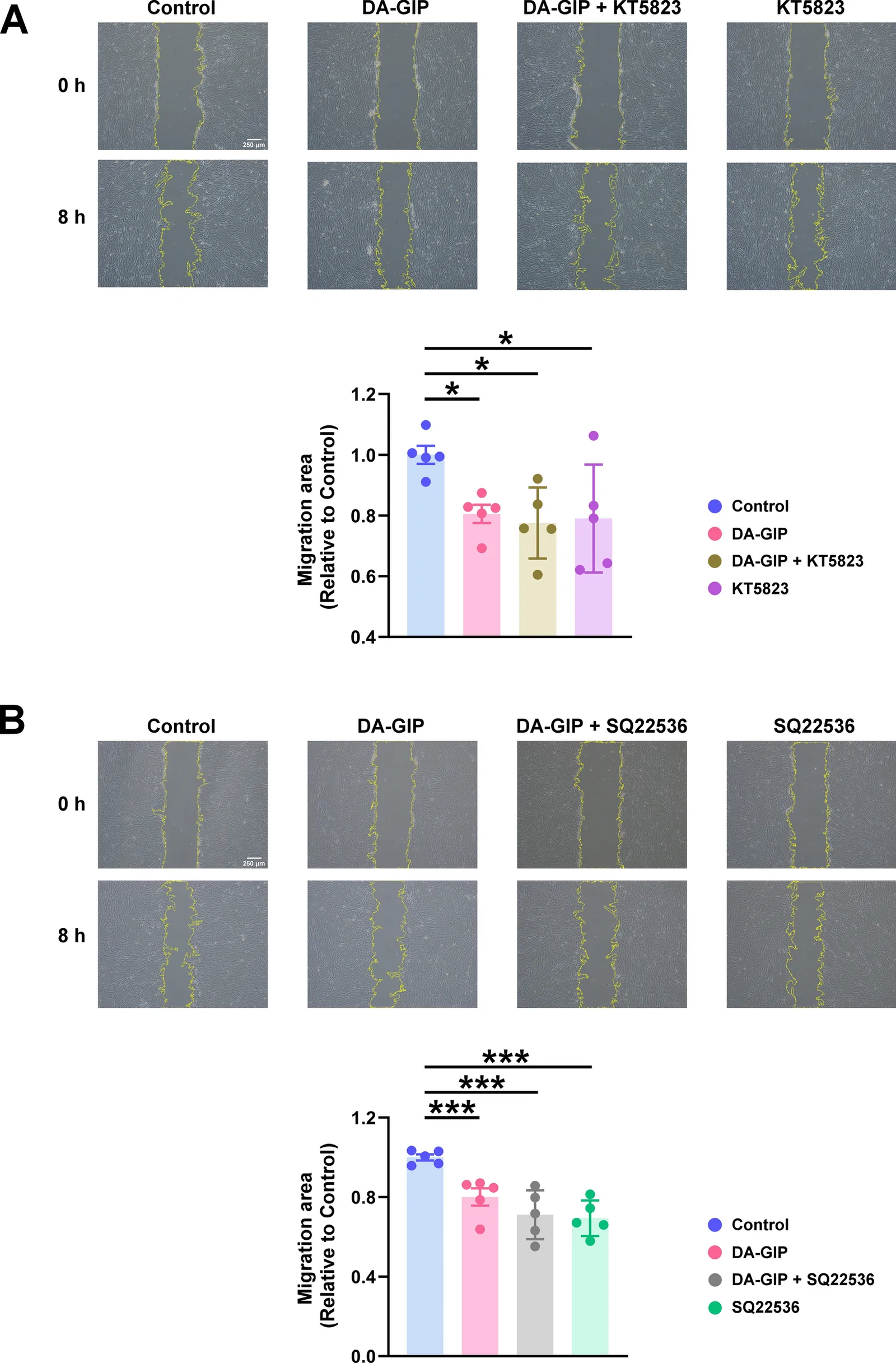
Effects of protein kinase G (PKG) and adenylyl cyclase inhibition on DA-GIP-induced suppression of cardiac fibroblast migration. Representative images (upper panel) and quantitative data (lower panel, *n* = 5) from a wound healing assay determining cell migration in control and DA-GIP (300 nM)-treated human cardiac fibroblasts in the presence or absence of **A** KT5823 (PKG inhibitor; 1 μM) or **B** SQ22536 (adenylyl cyclase inhibitor; 100 μM). Scale bar: 250 μm. ^*^p < 0.05 and ^***^p < 0.005

DA-GIP (300 nM)-treated fibroblasts exhibited significantly lower protein expression levels of TGF-β1 than did control fibroblasts (Fig. 2A). Consistent with this finding, TGF-β1 levels in the culture supernatants of DA-GIP (300 nM)-treated fibroblasts were significantly lower than those in the culture supernatants of control fibroblasts (Fig. 2B). We further investigated whether DA-GIP suppresses cardiac fibroblast activity through the cGMP/PKG pathway. DA-GIP (300 nM)-treated fibroblasts had significantly higher intracellular cGMP levels than did control fibroblasts (Fig. 2C). However, pretreatment with KT5823, a PKG inhibitor, did not attenuate the inhibitory effect of DA-GIP on cardiac fibroblast migration (Fig. 3A). Next, we investigated the role of cyclic adenosine monophosphate (cAMP) signaling in mediating the inhibitory effect of DA-GIP on fibroblast migration, given that GIP receptor activation may trigger downstream cAMP signaling. SQ22536-treated fibroblasts exhibited significantly lower migratory activity than did control fibroblasts (Fig. 3B). DA-GIP-treated, SQ22536-treated, and DA-GIP combined with SQ22536-treated fibroblasts exhibited similar levels of migratory activity. These findings suggest that the inhibitory effect of DA-GIP on fibroblast migration is independent of both the cGMP/PKG and cAMP pathways.

### Akt-dependent NO production mediates the antifibrotic effect of GIP receptor agonism

**Fig. 4:**
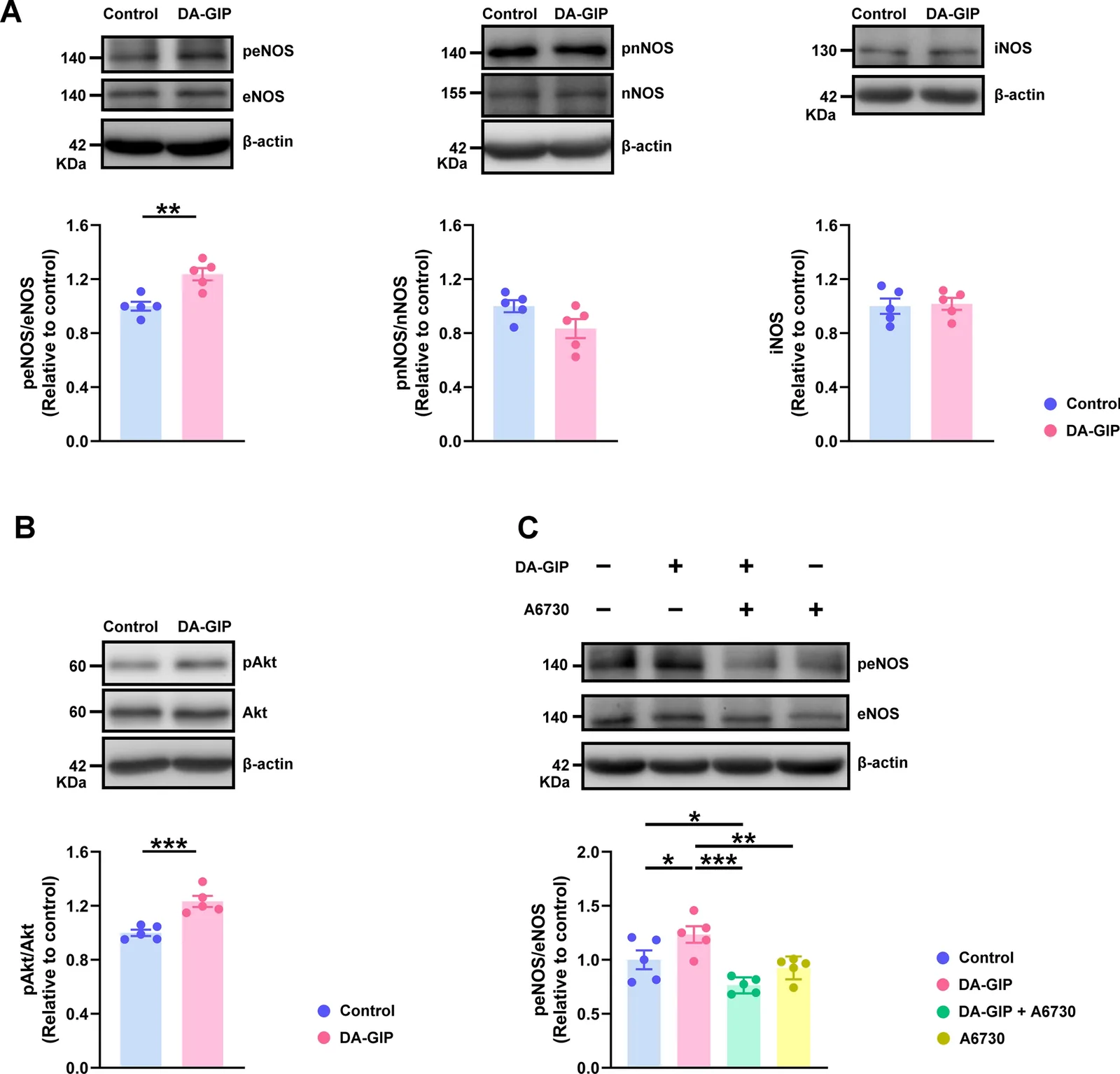
DA-GIP increases nitric oxide (NO) production through Akt-dependent endothelial NO synthase (eNOS) activation. **A** Representative immunoblots and quantitative data (*n* = 5) for total and phosphorylated (p) eNOS (left panel), neuronal NO synthase (nNOS; middle panel), and inducible NO synthase (iNOS; right panel) in control and DA-GIP (300 nM)-treated cardiac fibroblasts. **B** Representative immunoblots and quantitative data (*n* = 5) for total and phosphorylated Akt in control and DA-GIP (300 nM)-treated cardiac fibroblasts. **C** Representative immunoblots and quantitative data (*n* = 5) for eNOS phosphorylation in control and DA-GIP (300 nM)-treated fibroblasts in the presence or absence of A6730 (Akt inhibitor; 3 μM). ^*^p < 0.05, ^**^p < 0.01, and ^***^p < 0.005

NO is a principal stimulator of cGMP synthesis, and it exhibits antifibrotic activity. We investigated whether GIP receptor activation modulates NO production and examined the role of NO signaling in mediating the antifibrotic effect of DA-GIP. NO levels were significantly higher in the culture supernatants of DA-GIP (300 nM)-treated fibroblasts than in the culture supernatants of control fibroblasts (1.07 ± 0.08 vs. 0.55 ± 0.03 μM/10^6^ cells; *p* < 0.005). Furthermore, DA-GIP (300 nM)-treated fibroblasts had significantly higher phosphorylation levels of eNOS than did control fibroblasts (Fig. 4A). However, the levels of nNOS phosphorylation and iNOS protein expression were similar between control and DA-GIP-treated fibroblasts. DA-GIP (300 nM)-treated fibroblasts exhibited significantly higher levels of Akt phosphorylation than did control fibroblasts (Fig. 4B). Treatment with A6730, an Akt inhibitor, significantly attenuated the DA-GIP-induced increase in eNOS phosphorylation (Fig. 4C). These results suggest that DA-GIP increases NO production through the Akt-dependent activation of eNOS.

**Fig. 5:**
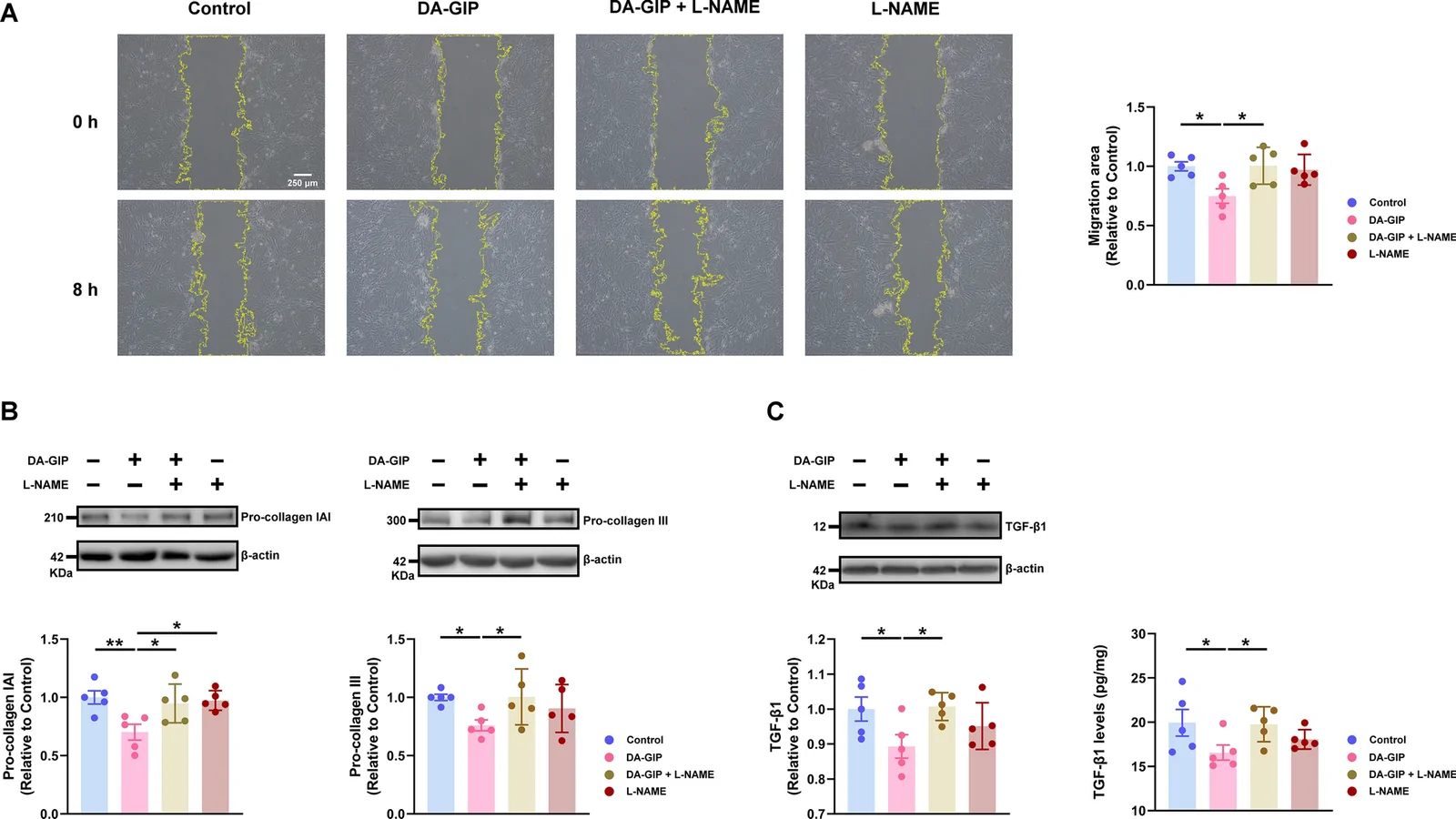
Nitric oxide (NO) mediates the suppressive effects of DA-GIP on human cardiac fibroblast migration, collagen production, and transforming growth factor (TGF)-β1 expression. **A** Representative images (left panel) and quantitative data (right panel, *n* = 5) from a wound healing assay assessing cell migration in control and DA-GIP (300 nM)-treated cardiac fibroblasts in the presence or absence of L-NAME (NO synthase inhibitor; 100 μM). Scale bar: 250 μm. **B** Representative immunoblots and quantitative data (*n* = 5) for pro-collagen IA1 (left panel) and pro-collagen III (right panel) expression in control and DA-GIP (300 nM)-treated cardiac fibroblasts in the presence or absence of L-NAME (100 μM). **C** Representative immunoblots and quantitative data (*n* = 5) for TGF-β1 expression in whole-cell lysates (left panel) and TGF-β1 levels in culture supernatants (right panel) from control and DA-GIP (300 nM)-treated cardiac fibroblasts in the presence or absence of L-NAME (100 μM). ^*^p < 0.05 and ^**^p < 0.01

We also found that DA-GIP (300 nM)-treated fibroblasts exhibited significantly lower migratory activity than did control fibroblasts and DA-GIP combined with L-NAME-treated fibroblasts (Fig. 5A). However, migratory activity was similar among control, L-NAME-treated, and DA-GIP combined with L-NAME–treated fibroblasts. Furthermore, DA-GIP (300 nM)-treated fibroblasts exhibited significantly lower expression levels of pro-collagen IA1 and pro-collagen III proteins than did control and DA-GIP combined with L-NAME–treated fibroblasts (Fig. 5B). The expression levels of both pro-collagen IA1 and pro-collagen III proteins were similar among control, L-NAME-treated, and DA-GIP combined with L-NAME–treated fibroblasts. TGF-β1 levels in whole-cell lysates and culture supernatants were significantly lower for DA-GIP (300 nM)-treated fibroblasts than for control and DA-GIP combined with L-NAME–treated fibroblasts (Fig. 5C). These findings suggest that NO mediates the inhibitory effect of DA-GIP on cardiac fibroblast activity.

### GIP receptor activation improves cardiac function and suppresses myocardial fibrosis in isoproterenol-induced heart failure

**Fig. 6:**
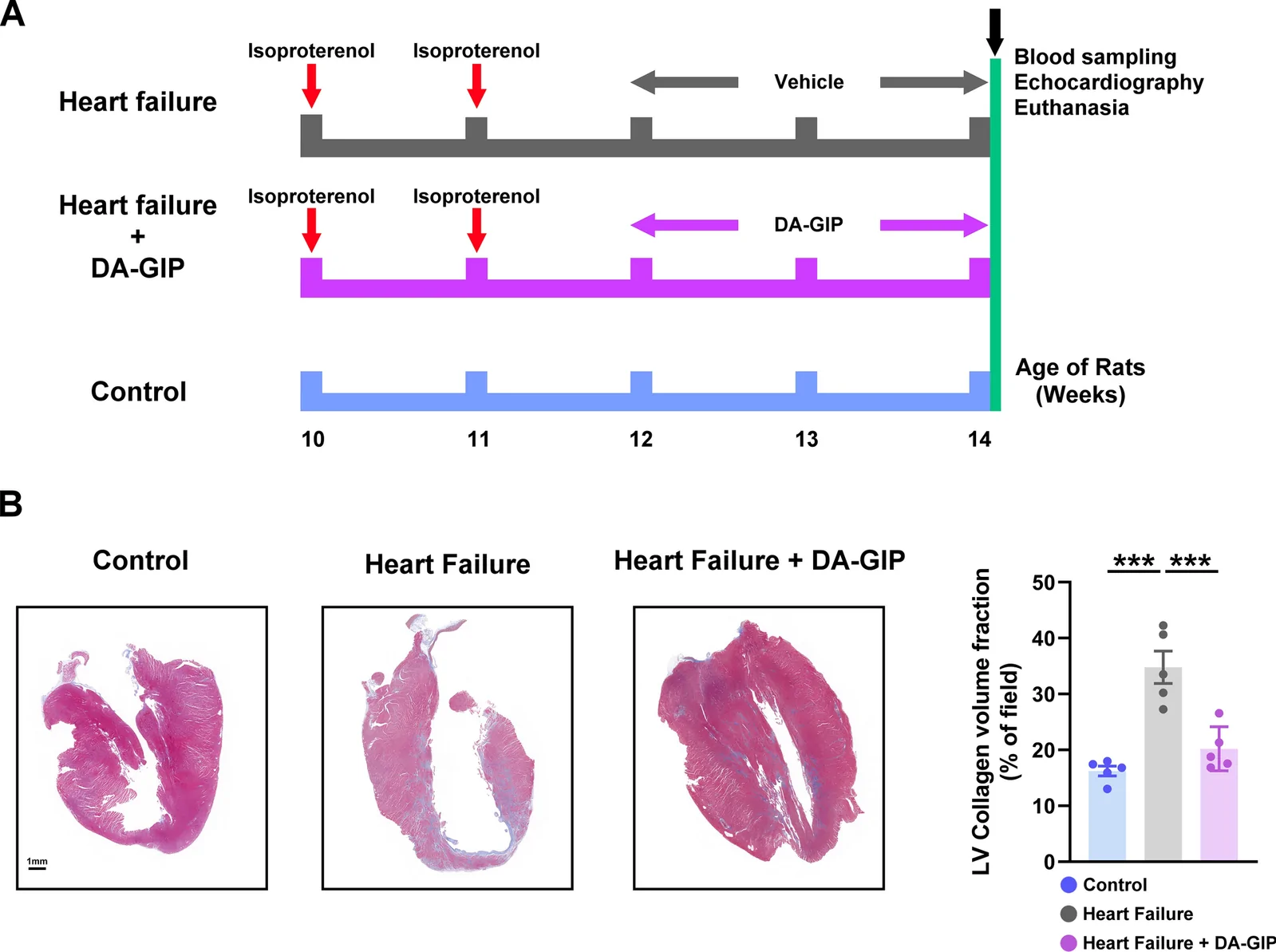
Effects of DA-GIP on left ventricular (LV) myocardial fibrosis in rats with isoproterenol-induced heart failure. **A** Schematic overview of the experimental protocol for control rats and rats with isoproterenol-induced heart failure (100 mg/kg; subcutaneous injection, once a week for 2 weeks) treated with vehicle (0.9% normal saline) or DA-GIP (24 nM/kg; subcutaneous injection, twice a day for 2 weeks). **B** Representative images of Masson’s trichrome–stained LV sections (left panel) and quantitative data for collagen volume fraction (right panel) from control rats (*n* = 5) and heart failure rats treated with DA-GIP (*n* = 5) or vehicle (*n* = 5). Fibrotic areas are stained blue. ^***^p < 0.005

**Fig. 7:**
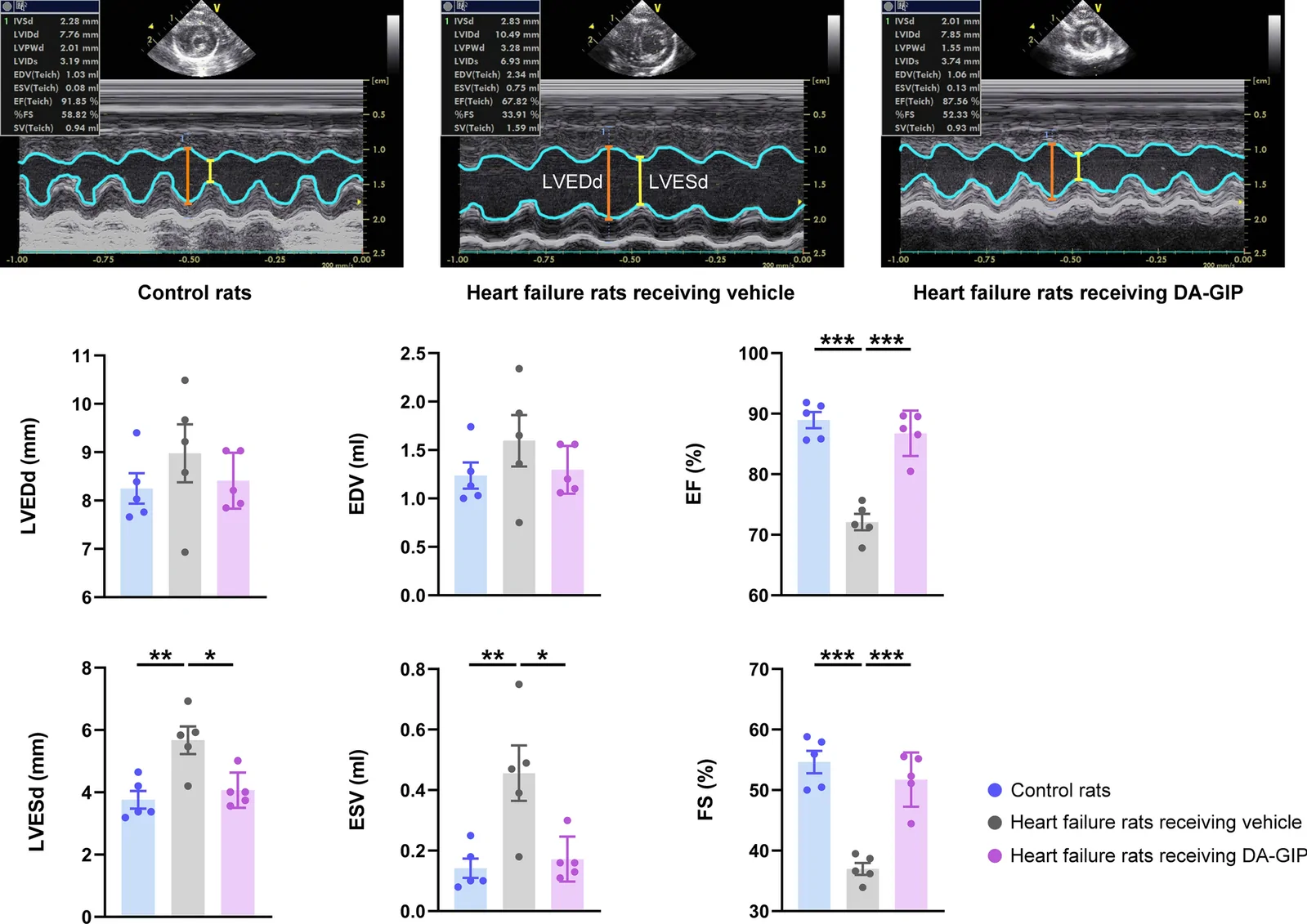
Effects of DA-GIP on cardiac structure and systolic function in rats with isoproterenol-induced heart failure. Representative echocardiographic images and quantitative measurements of left ventricular (LV) end-diastolic diameter (LVEDd), LV end-systolic diameter (LVESd), LV end-diastolic volume (EDV), LV end-systolic volume (ESV), ejection fraction (EF), and fractional shortening (FS) in control rats (n = 5) and heart failure rats treated with DA-GIP (n = 5) or vehicle (n = 5). ^*^p < 0.05, ^**^p < 0.01, and ^***^p < 0.005

We assessed the effects of GIP receptor agonism on cardiac structure, function, and fibrosis in rats with isoproterenol-induced heart failure (Fig. 6A). As shown in Table 1, body weight, heart rate, and serum biochemical parameters (creatinine, aspartate transaminase, alanine transaminase, fasting glucose, insulin, total cholesterol, triglycerides, and high-density lipoprotein cholesterol levels) were similar among control, heart failure, and DA-GIP-treated heart failure rats. However, heart weight and heart weight/body weight ratio were significantly higher in heart failure rats than in control rats. DA-GIP-treated heart failure rats exhibited a trend toward lower heart weight and heart weight/body weight ratio than did untreated heart failure rats. Masson’s trichrome staining revealed a significantly higher ventricular collagen volume fraction, indicating more severe myocardial fibrosis, in heart failure rats than in control and DA-GIP-treated heart failure rats (Fig. 6B). Collagen volume fraction was similar between control and DA-GIP-treated heart failure rats. Furthermore, echocardiography indicated that LV end-diastolic diameter and end-diastolic volume were similar among control, heart failure, and DA-GIP-treated heart failure rats (Fig. 7). However, heart failure rats exhibited significantly larger LV end-systolic diameter and end-systolic volume and lower ejection fraction and fractional shortening than did control and DA-GIP-treated heart failure rats. These systolic functional parameters were similar between control and DA-GIP-treated heart failure rats.

**Table 1:** Physical and biochemical characteristics of control, heart failure, and DA-GIP-treated heart failure rats

	Control	Heart failure	Heart failure + DA-GIP
Body weight (g)	510 ± 10.8	501.2 ± 9.3	509.6 ± 9.4
Heart weight (g)	1.7 ± 0.0	2.4 ± 0.3^*^	2.0 ± 0.1
Heart weight/body weight (mg/g)	3.3 ± 0.1	4.7 ± 0.6^*^	4.0 ± 0.2
Heart rate (bpm)	398.5 ± 7.3	386.8 ± 15.2	366.1 ± 18.1
Glucose (mg/dL)	187.2 ± 12.1	201.4 ± 20.0	204.6 ± 14.3
Insulin (ng/mL)	1.2 ± 0.3	1.8 ± 0.3	2.0 ± 0.8
Creatinine (mg/dL)	0.26 ± 0.0	0.28 ± 0.0	0.27 ± 0.0
Aspartate transaminase (U/L)	77.0 ± 4.6	72.4 ± 5.7	69.4 ± 7.3
Alanine transaminase (U/L)	31.0 ± 2.3	27.6 ± 0.9	31.2 ± 3.8
Total cholesterol (mg/dL)	57.2 ± 3.9	57.0 ± 3.4	61.4 ± 6.5
Triglycerides (mg/dL)	98.8 ± 6.3	81.6 ± 3.1	88.4 ± 11.5
High-density lipoprotein cholesterol (mg/dL)	33.4 ± 4.0	34.4 ± 4.4	41.8 ± 6.5

Data are presented as mean ± standard error of the mean values

Five rats were used per group

Significance: ^*^p < 0.05, compared with the control

## Discussion

**Fig. 8:**
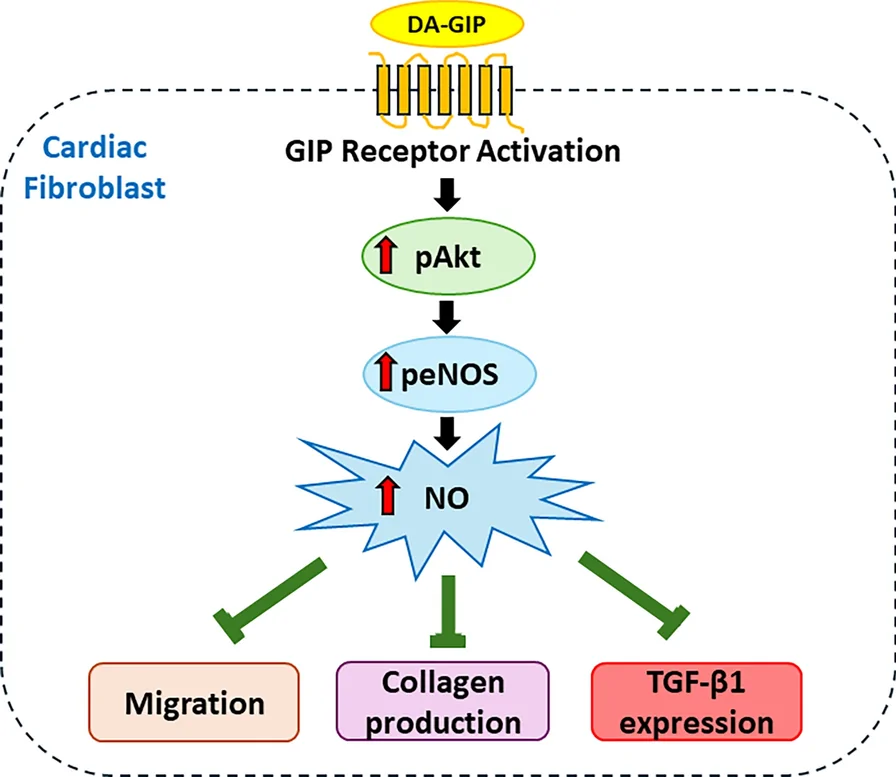
Proposed mechanism by which DA-GIP suppresses cardiac fibroblast activity. DA-GIP activates the glucose-dependent insulinotropic polypeptide (GIP) receptor, leading to Akt-dependent endothelial nitric oxide synthase (eNOS) activation and increased nitric oxide (NO) production. Upregulated NO signaling inhibits cardiac fibroblast migration, collagen production, and transforming growth factor (TGF)-β1 expression, thereby suppressing cardiac fibrogenesis

GIP receptor agonists were primarily developed for the treatment of DM and obesity. Both obesity and hyperglycemia promote cardiac fibrogenesis [28]. In the present study, DA-GIP treatment did not significantly alter body weight or serum insulin, glucose, or lipid level in heart failure rats. Therefore, the antifibrotic effect of DA-GIP is less likely to be mediated through metabolic modulation. We found that pharmacological activation of the GIP receptor directly suppressed cardiac fibroblast activity. As summarized in Fig. 8, DA-GIP treatment significantly reduced fibroblast migration, collagen production, and TGF-β1 expression. DA-GIP promoted Akt-dependent eNOS activation, thereby enhancing NO production and mediating its antifibrotic effects. Consistent with these findings in cultured cardiac fibroblasts, DA-GIP reduced LV myocardial fibrosis, improved chamber dilatation, and mitigated systolic dysfunction in heart failure rats. These findings highlight GIP receptor agonism as a promising therapeutic strategy for cardiac fibrosis.

The cGMP/PKG pathway is a key signaling cascade that suppresses cardiac fibrosis. Our study revealed that DA-GIP significantly increased cellular cGMP levels in human cardiac fibroblasts. However, inhibition of PKG with KT5823 did not attenuate the inhibitory effect of DA-GIP on cardiac fibroblast migration. Therefore, the antifibrotic effect of DA-GIP is not mediated by the cGMP/PKG pathway. Additionally, the GIP receptor is a seven-transmembrane class B type G protein-coupled receptor whose activation primarily stimulates adenylyl cyclase, thereby increasing intracellular cAMP levels [16]. Native GIP and GIP receptor agonists can activate cAMP signaling, which plays a vital role in regulating cardiac fibrogenesis [12, 15]. Activation of adenylyl cyclase was demonstrated to inhibit TGF-β-induced myofibroblast transformation and collagen synthesis in adult rat cardiac fibroblasts [29]. Increasing cellular cAMP levels reversed multiple features of the TGF-β1-induced myofibroblastic phenotype in rat cardiac fibroblasts [30]. However, in our study, inhibition of adenylyl cyclase with SQ22536 did not impair the inhibitory effect of DA-GIP on cardiac fibroblast migration. Similar to DA-GIP-treated fibroblasts, SQ22536-treated cardiac fibroblasts exhibited significantly lower migratory activity than did control fibroblasts. Overall, these findings suggest that the inhibitory effect of DA-GIP on cardiac fibroblast activity is not mediated through the activation of cAMP signaling.

eNOS and nNOS are constitutively expressed in the cardiovascular system and generally confer protection against fibrosis, whereas iNOS is induced under inflammatory and oxidative stress conditions and typically promotes fibrotic remodeling [31]. In isolated neonatal rat cardiac fibroblasts, inhibition of eNOS, but not nNOS or iNOS, increased collagen synthesis and fibroblast migration [32], suggesting that eNOS is a central regulator of cardiac fibroblast activity. In the present study, the levels of nNOS phosphorylation and iNOS protein expression were similar between control and DA-GIP (300 nM)-treated fibroblasts. These findings indicate that nNOS and iNOS do not mediate the antifibrotic effect of DA-GIP on cardiac fibroblasts. GIP activated eNOS and promoted NO release in human umbilical vein endothelial cells, leading to a protective effect against peripheral arterial remodeling in mice [33]. Consistent with these findings, we found that DA-GIP-treated human cardiac fibroblasts had significantly higher NO levels than did control fibroblasts. We further noted that DA-GIP activated eNOS, but not nNOS, in human cardiac fibroblasts. Therefore, DA-GIP increases NO production through eNOS activation, thereby inhibiting cardiac fibroblast activity independently of PKG. Although eNOS is a calcium-dependent enzyme, DA-GIP is unlikely to activate eNOS by increasing intracellular calcium levels, given that elevated intracellular calcium levels promote cardiac fibroblast activity. Moreover, Akt, a serine/threonine protein kinase, directly phosphorylates and activates eNOS, thereby increasing NO production [34]. In the present study, DA-GIP significantly increased Akt phosphorylation, and Akt inhibition attenuated the DA-GIP-induced activation of eNOS in human cardiac fibroblasts. Furthermore, inhibition of eNOS with L-NAME significantly attenuated the inhibitory effects of DA-GIP on human cardiac fibroblast migration, collagen production, and TGF-β1 expression. These findings suggest that DA-GIP increases NO production through Akt-dependent activation of eNOS, thereby exerting its antifibrotic effects.

This study has several limitations. The results were obtained from cellular and animal models, and their clinical applicability remains to be established. Furthermore, GIP receptor agonism may reduce cardiac workload by increasing NO production, thereby promoting vasodilation and improving myocardial compliance. Future studies should incorporate pressure–volume loop analysis to further clarify the effects of GIP receptor agonism on ventricular mechanics in heart failure.

## Conclusion

Activation of the GIP receptor suppresses cardiac fibroblast activity and improves cardiac remodeling and function in experimental heart failure, likely through the Akt-dependent activation of NO signaling.

## Data Availability

The datasets used and analyzed in the present study are available from the corresponding author upon reasonable request.

## Abbreviations

α-SMA: α-smooth muscle actin
cAMP: Cyclic adenosine monophosphate
cGMP: Cyclic guanosine monophosphate
DA-GIP: [D-Ala^2^] glucose-dependent insulinotropic polypeptide
DM: Diabetes mellitus
EdU: 5-ethynyl-2′-deoxyuridine
eNOS: Endothelial NOS
GIP: Glucose-dependent insulinotropic polypeptide
GLP-1: Glucagon-like peptide-1
iNOS: Inducible NOS
L-NAME: *N*^*ω*^-nitro-L-arginine methyl ester
LV: Left ventricular
nNOS: Neuronal NOS
NO: Nitric oxide
NOS: NO synthase
PKG: Protein kinase G
TGF-β1: Transforming growth factor-β1

## References

[1] Hammoud R, Drucker DJ. Beyond the pancreas: contrasting cardiometabolic actions of GIP and GLP1. Nat Rev Endocrinol. 2023;19(4):201–16. 36509857 10.1038/s41574-022-00783-3

[2] Müller TD, Adriaenssens A, Ahrén B, Blüher M, Birkenfeld AL, Campbell JE, et al. Glucose-dependent insulinotropic polypeptide (GIP). Mol Metab. 2025;95:102118. 40024571 10.1016/j.molmet.2025.102118 PMC11931254

[3] Wolfe MM, Boylan MO, Chin WW. Glucose-dependent insulinotropic polypeptide in incretin physiology: role in health and disease. Endocr Rev. 2025;46(4):479–500. 39951489 10.1210/endrev/bnaf006 PMC12259236

[4] Nauck MA, D’Alessio DA. Tirzepatide, a dual GIP/GLP-1 receptor co-agonist for the treatment of type 2 diabetes with unmatched effectiveness regrading glycaemic control and body weight reduction. Cardiovasc Diabetol. 2022;21(1):169. 36050763 10.1186/s12933-022-01604-7 PMC9438179

[5] Aronne LJ, Horn DB, le Roux CW, Ho W, Falcon BL, Gomez Valderas E, et al. Tirzepatide as compared with semaglutide for the treatment of obesity. N Engl J Med. 2025;393(1):26–36. 40353578 10.1056/NEJMoa2416394

[6] Greenwell AA, Chahade JJ, Ussher JR. Cardiovascular biology of the GIP receptor. Peptides. 2020;125:170228. 31812593 10.1016/j.peptides.2019.170228

[7] Karhunen V, Daghlas I, Zuber V, Vujkovic M, Olsen AK, Knudsen LB, et al. Leveraging human genetic data to investigate the cardiometabolic effects of glucose-dependent insulinotropic polypeptide signalling. Diabetologia. 2021;64(12):2773–8. 34505161 10.1007/s00125-021-05564-7 PMC8563538

[8] Kahles F, Rückbeil MV, Arrivas MC, Mertens RW, Moellmann J, Biener M, et al. Association of glucose-dependent insulinotropic polypeptide levels with cardiovascular mortality in patients with acute myocardial infarction. J Am Heart Assoc. 2021;10(13):e019477. 34212758 10.1161/JAHA.120.019477 PMC8403319

[9] Lighthouse JK, Small EM. Transcriptional control of cardiac fibroblast plasticity. J Mol Cell Cardiol. 2016;91:52–60. 26721596 10.1016/j.yjmcc.2015.12.016 PMC4764462

[10] Travers JG, Kamal FA, Robbins J, Yutzey KE, Blaxall BC. Cardiac fibrosis: the fibroblast awakens. Circ Res. 2016;118(6):1021–40. 26987915 10.1161/CIRCRESAHA.115.306565 PMC4800485

[11] Nagalingam RS, Safi HA, Czubryt MP. Gaining myocytes or losing fibroblasts: challenges in cardiac fibroblast reprogramming for infarct repair. J Mol Cell Cardiol. 2016;93:108–14. 26640115 10.1016/j.yjmcc.2015.11.029

[12] Hiromura M, Mori Y, Kohashi K, Terasaki M, Shinmura K, Negoro T, et al. Suppressive effects of glucose-dependent insulinotropic polypeptide on cardiac hypertrophy and fibrosis in angiotensin II-infused mouse models. Circ J. 2016;80(9):1988–97. 27375170 10.1253/circj.CJ-16-0152

[13] Hiromura M, Mori Y, Terasaki M, Kushima H, Saito T, Osaka N, et al. Glucose-dependent insulinotropic polypeptide inhibits cardiac hypertrophy and fibrosis in diabetic mice via suppression of TGF-β2. Diab Vasc Dis Res. 2021;18(2):1479164121999034. 35012372 10.1177/1479164121999034 PMC8755933

[14] Dani SS, Makwana B, Khadke S, Kumar A, Jhund P, Nasir K, et al. An observational study of cardiovascular outcomes of tirzepatide vs glucagon-like peptide-1 receptor agonists. JACC Adv. 2025;4(5):101740. 40447342 10.1016/j.jacadv.2025.101740 PMC12235410

[15] Ussher JR, Campbell JE, Mulvihill EE, Baggio LL, Bates HE, McLean BA, et al. Inactivation of the glucose-dependent insulinotropic polypeptide receptor improves outcomes following experimental myocardial infarction. Cell Metab. 2018;27(2):450-60.e6. 29275960 10.1016/j.cmet.2017.11.003

[16] Heimbürger SM, Bergmann NC, Augustin R, Gasbjerg LS, Christensen MB, Knop FK. Glucose-dependent insulinotropic polypeptide (GIP) and cardiovascular disease. Peptides. 2020;125:170174. 31689454 10.1016/j.peptides.2019.170174

[17] Tsai EJ, Kass DA. Cyclic GMP signaling in cardiovascular pathophysiology and therapeutics. Pharmacol Ther. 2009;122(3):216–38. 19306895 10.1016/j.pharmthera.2009.02.009 PMC2709600

[18] Inserte J, Garcia-Dorado D. The cGMP/PKG pathway as a common mediator of cardioprotection: translatability and mechanism. Br J Pharmacol. 2015;172(8):1996–2009. 25297462 10.1111/bph.12959 PMC4386977

[19] Li P, Wang D, Lucas J, Oparil S, Xing D, Cao X, et al. Atrial natriuretic peptide inhibits transforming growth factor beta-induced Smad signaling and myofibroblast transformation in mouse cardiac fibroblasts. Circ Res. 2008;102(2):185–92. 17991884 10.1161/CIRCRESAHA.107.157677

[20] Chen J, Shearer GC, Chen Q, Healy CL, Beyer AJ, Nareddy VB, et al. Omega-3 fatty acids prevent pressure overload-induced cardiac fibrosis through activation of cyclic GMP/protein kinase G signaling in cardiac fibroblasts. Circulation. 2011;123(6):584–93. 21282499 10.1161/CIRCULATIONAHA.110.971853 PMC3056077

[21] Hou J, Kato H, Cohen RA, Chobanian AV, Brecher P. Angiotensin II-induced cardiac fibrosis in the rat is increased by chronic inhibition of nitric oxide synthase. J Clin Invest. 1995;96(5):2469–77. 7593636 10.1172/JCI118305 PMC185900

[22] Cai Z, Wu C, Xu Y, Cai J, Zhao M, Zu L. The NO-cGMP-PKG axis in HFpEF: from pathological mechanisms to potential therapies. Aging Dis. 2023;14(1):46–62. 36818566 10.14336/AD.2022.0523 PMC9937694

[23] Garg UC, Hassid A. Nitric oxide-generating vasodilators inhibit mitogenesis and proliferation of BALB/C 3T3 fibroblasts by a cyclic GMP-independent mechanism. Biochem Biophys Res Commun. 1990;171(1):474–9. 1697465 10.1016/0006-291x(90)91417-q

[24] Hinke SA, Gelling RW, Pederson RA, Manhart S, Nian C, Demuth HU, et al. Dipeptidyl peptidase IV-resistant [D-Ala(2)]glucose-dependent insulinotropic polypeptide (GIP) improves glucose tolerance in normal and obese diabetic rats. Diabetes. 2002;51(3):652–61. 11872663 10.2337/diabetes.51.3.652

[25] Lee TW, Lee TI, Higa S, Kao YH, Chen YJ. Calcitriol deficiency reduces myocardial ATP production with disturbed mitochondrial dynamics and increased uncoupling protein 2 expression. Eur J Pharmacol. 2025;1002:177858. 40553779 10.1016/j.ejphar.2025.177858

[26] Lee TW, Chung CC, Lee TI, Lin YK, Kao YH, Chen YJ. Fibroblast growth factor 23 stimulates cardiac fibroblast activity through phospholipase C-mediated calcium signaling. Int J Mol Sci. 2021. 10.3390/ijms23010166. 35008591 PMC8745152

[27] Chung CC, Lin YK, Chen YC, Kao YH, Yeh YH, Trang NN, et al. Empagliflozin suppressed cardiac fibrogenesis through sodium-hydrogen exchanger inhibition and modulation of the calcium homeostasis. Cardiovasc Diabetol. 2023;22(1):27. 36747205 10.1186/s12933-023-01756-0 PMC9903522

[28] Cavalera M, Wang J, Frangogiannis NG. Obesity, metabolic dysfunction, and cardiac fibrosis: pathophysiological pathways, molecular mechanisms, and therapeutic opportunities. Transl Res. 2014;164(4):323–35. 24880146 10.1016/j.trsl.2014.05.001 PMC4180761

[29] Swaney JS, Roth DM, Olson ER, Naugle JE, Meszaros JG, Insel PA. Inhibition of cardiac myofibroblast formation and collagen synthesis by activation and overexpression of adenylyl cyclase. Proc Natl Acad Sci. 2005;102(2):437–42. 15625103 10.1073/pnas.0408704102 PMC544320

[30] Lu D, Aroonsakool N, Yokoyama U, Patel HH, Insel PA. Increase in cellular cyclic AMP concentrations reverses the profibrogenic phenotype of cardiac myofibroblasts: a novel therapeutic approach for cardiac fibrosis. Mol Pharmacol. 2013;84(6):787–93. 24085841 10.1124/mol.113.087742 PMC3834140

[31] Umar S, van der Laarse A. Nitric oxide and nitric oxide synthase isoforms in the normal, hypertrophic, and failing heart. Mol Cell Biochem. 2010;333(1):191–201. 19618122 10.1007/s11010-009-0219-x

[32] Kazakov A, Hall R, Jagoda P, Bachelier K, Müller-Best P, Semenov A, et al. Inhibition of endothelial nitric oxide synthase induces and enhances myocardial fibrosis. Cardiovasc Res. 2013;100(2):211–21. 23863197 10.1093/cvr/cvt181

[33] Mori Y, Kushima H, Koshibu M, Saito T, Hiromura M, Kohashi K, et al. Glucose-dependent insulinotropic polypeptide suppresses peripheral arterial remodeling in male mice. Endocrinology. 2018;159(7):2717–32. 29846588 10.1210/en.2018-00336

[34] Fulton D, Gratton JP, McCabe TJ, Fontana J, Fujio Y, Walsh K, et al. Regulation of endothelium-derived nitric oxide production by the protein kinase Akt. Nature. 1999;399(6736):597–601. 10376602 10.1038/21218 PMC3637917

